# Hexaaqua­copper(II) dichloride bis­(hexa­methyl­enetetra­mine) tetra­hydrate

**DOI:** 10.1107/S1600536808020916

**Published:** 2008-07-12

**Authors:** Zhan Lin Li, Xin Jian Yao, Wen Wu, Ya Wen Xuan

**Affiliations:** a Department of Chemistry, Zhou Kou Normal University, Zhou Kou 466001, People’s Republic of China

## Abstract

The title compound, [Cu(H_2_O)_6_]Cl_2_·2C_6_H_12_N_4_·4H_2_O, was prepared under mild hydro­thermal conditions. The asymmetric unit consists of one half of the [Cu(H_2_O)_6_]^2+^ cation, a hexa­methyl­enetetra­mine mol­ecule, two solvent water mol­ecules and a chloride ion. The formula unit is generated by crystallographic inversion symmetry. The Cu atom lies on a crystallographic inversion centre. It is in a slightly distorted octa­hedral coordination environment. In the crystal structure, inter­molecular O—H⋯O, O—H⋯N and O—H⋯Cl hydrogen bonds link the components into a three-dimensional network.

## Related literature

For a related structure, see: Kinzhibalo *et al.* (2002 ▶).
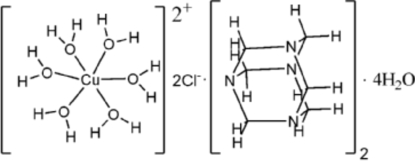


## Experimental

### 

#### Crystal data

[Cu(H_2_O)_6_]Cl_2_·2C_6_H_12_N_4_·4H_2_O
*M* *_r_* = 594.99
Triclinic, 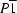

*a* = 9.321 (3) Å
*b* = 9.3923 (16) Å
*c* = 9.4261 (16) Å
α = 119.523 (2)°
β = 94.153 (3)°
γ = 101.065 (3)°
*V* = 691.1 (3) Å^3^
*Z* = 1
Mo *K*α radiation
μ = 1.04 mm^−1^
*T* = 291 (2) K
0.36 × 0.29 × 0.15 mm

#### Data collection

Bruker SMART CCD diffractometer
Absorption correction: multi-scan (*SADABS*; Sheldrick, 1996 ▶) *T*_min_ = 0.709, *T*_max_ = 0.860
5328 measured reflections
2551 independent reflections
2083 reflections with *I* > 2σ(*I*)
*R*_int_ = 0.027

#### Refinement

*R*[*F* ^2^ > 2σ(*F* ^2^)] = 0.041
*wR*(*F* ^2^) = 0.108
*S* = 1.05
2551 reflections
151 parameters
H-atom parameters constrained
Δρ_max_ = 0.30 e Å^−3^
Δρ_min_ = −0.51 e Å^−3^

### 

Data collection: *SMART* (Bruker, 2002 ▶); cell refinement: *SAINT* (Bruker, 2002 ▶); data reduction: *SAINT*; program(s) used to solve structure: *SHELXS97* (Sheldrick, 2008 ▶); program(s) used to refine structure: *SHELXL97* (Sheldrick, 2008 ▶); molecular graphics: *SHELXTL* (Sheldrick, 2008 ▶); software used to prepare material for publication: *SHELXTL*.

## Supplementary Material

Crystal structure: contains datablocks I, global. DOI: 10.1107/S1600536808020916/lh2645sup1.cif

Structure factors: contains datablocks I. DOI: 10.1107/S1600536808020916/lh2645Isup2.hkl

Additional supplementary materials:  crystallographic information; 3D view; checkCIF report

## Figures and Tables

**Table:** 

Cu1—O2	2.017 (2)
Cu1—O1	2.045 (2)
Cu1—O3	2.053 (2)

**Table:** 

O2—Cu1—O2^i^	180
O2—Cu1—O1	87.24 (9)
O2—Cu1—O1^i^	92.76 (9)
O1—Cu1—O1^i^	180
O2—Cu1—O3^i^	90.30 (10)
O1—Cu1—O3^i^	86.64 (9)
O2—Cu1—O3	89.70 (10)
O1—Cu1—O3	93.36 (9)
O3^i^—Cu1—O3	180

Symmetry code: (i) 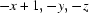.

**Table 2:** Hydrogen-bond geometry (Å, °)

*D*—H⋯*A*	*D*—H	H⋯*A*	*D*⋯*A*	*D*—H⋯*A*
O1—H1*W*⋯N3	0.82	2.04	2.814 (3)	158
O1—H2*W*⋯O5^ii^	0.83	1.94	2.734 (3)	162
O2—H3*W*⋯N2^iii^	0.83	1.99	2.800 (3)	167
O2—H4*W*⋯O4^iii^	0.83	1.89	2.700 (3)	165
O3—H5*W*⋯Cl1	0.82	2.54	3.190 (2)	137
O3—H6*W*⋯N1^iv^	0.82	2.00	2.805 (3)	165
O4—H7*W*⋯Cl1	0.83	2.35	3.170 (3)	168
O4—H8*W*⋯N4^v^	0.84	2.00	2.829 (4)	174
O5—H9*W*⋯Cl1	0.83	2.43	3.245 (3)	169
O5—H10*W*⋯Cl1^vi^	0.83	2.37	3.200 (3)	175

Symmetry codes: (ii) 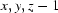; (iii) 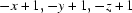; (iv) 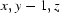; (v) 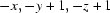; (vi) 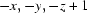.

**Fig. 1.**
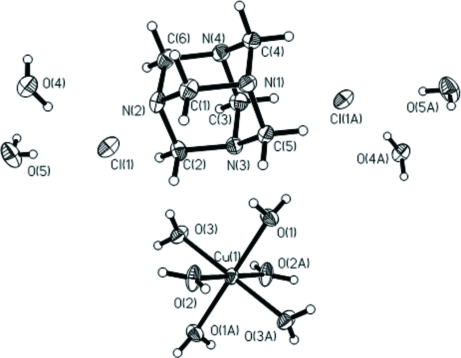
The asymmetric unit and symmetry related atoms of the title compound with 30% probability ellipsoids [symmetry code: (A) -x+1, -y, -z].

**Fig. 2.**
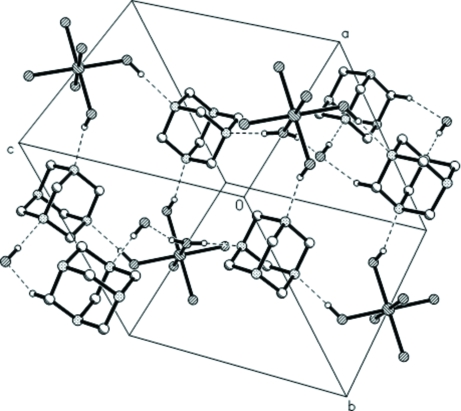
Hydrogen bonding [dashed lines] in part of the crystal structure between [Cu(H2O)6]2+ cations, hexamethylenetetramine molecules and water molecules.

**Fig. 3.**
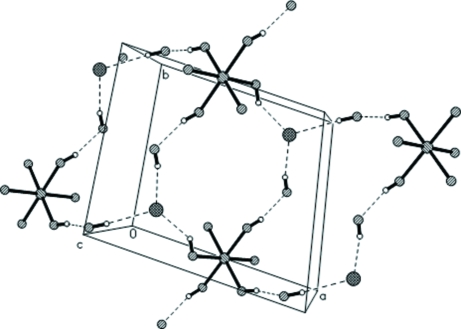
Hydrogen bonding [dashed lines] in part of the crystal structure between the [Cu(H2O)6]2+ cations, chloride anions and water molecules.

## supplementary crystallographic information

### Comment

The asymmetric unit and some symmetry related atoms are shown in Fig.1. The asymmetric unit consists of one half of hexaaqua Cu^II^ cation, one chloride anion, one uncoordinated neutral hexamethylenetetramine molecule and two molecules of water of crystallization. In the crystal structure, hydrogen bonding between [Cu(H_2_O)_6_]^2+^ cations and hexamethylenetetramine molecules, and those between [Cu(H_2_O)_6_]^2+^ cations and chloride ions are shown in Fig. 2 and Fig.3, respectively. A 16-membered ring formed by cations and hexamethylenetetramine moieties *via* the H-bonding interactions propagates along the *c*-axis. The chloride ion H-bonded with the uncoordinated water molecules gives rise to a number of anionic ring systems (Fig. 3). One of the hydrogen atoms of the uncoordinated water molecule connects the chloride ion and forms a 16-membered ring. The combonation of these anionic and cationic frameworks results in the formation of a three-dimensional network.

### Experimental

All reagents were of AR grade and used without further purification. C_6_H_12_N_4_ (1.401 g, 10 mmol) was dissolved in 50 ml EtOH/H_2_O (V:V = 1:1) solution, then the resultant solution was added in 10 ml double-distilled water containing CuCl_2_.2H_2_O (0.171 g, 1 mmol), The resulting solution was heated at 373 K for 96 h. After cooling to room temperature, blue crystals were obtained in a yield up to 48.6%.

### Refinement

H atoms bonded to O atoms were located in a difference map and included in their 'as found' positions with *U*_iso_(H) = 1.5U_eq_(O). Other H atoms were positioned geometrically with C-H = 0.97 Å and with *U*_iso_(H)=1.2*U*_eq_(C). All H atoms were treated as riding.

### Crystal data

**Table:**

[Cu(H_2_O)_6_]Cl_2_·2C_6_H_12_N_4_·4H_2_O	*Z* = 1
*M**_r_* = 594.99	*F*_000_ = 315
Triclinic, *P*1	*D*_x_ = 1.430 Mg m^−^^3^
Hall symbol: -P 1	Mo *K*α radiation λ = 0.71073 Å
*a* = 9.321 (3) Å	Cell parameters from 1415 reflections
*b* = 9.3923 (16) Å	θ = 2.5–22.9º
*c* = 9.4261 (16) Å	µ = 1.04 mm^−^^1^
α = 119.523 (2)º	*T* = 291 (2) K
β = 94.153 (3)º	Block, blue
γ = 101.065 (3)º	0.36 × 0.29 × 0.15 mm
*V* = 691.1 (3) Å^3^	

### Data collection

**Table:**

Bruker SMART CCD diffractometer	2551 independent reflections
Radiation source: fine-focus sealed tube	2083 reflections with *I* > 2σ(*I*)
Monochromator: graphite	*R*_int_ = 0.027
Detector resolution: 0 pixels mm^-1^	θ_max_ = 25.5º
*T* = 291(2) K	θ_min_ = 2.5º
φ and ω scans	*h* = −11→11
Absorption correction: multi-scan(SADABS; Sheldrick, 1996)	*k* = −11→11
*T*_min_ = 0.709, *T*_max_ = 0.860	*l* = −11→11
5328 measured reflections	

### Refinement

**Table:**

Refinement on *F*^2^	Secondary atom site location: difference Fourier map
Least-squares matrix: full	Hydrogen site location: inferred from neighbouring sites
*R*[*F*^2^ > 2σ(*F*^2^)] = 0.041	H-atom parameters constrained
*wR*(*F*^2^) = 0.108	*w* = 1/[σ^2^(*F*_o_^2^) + (0.0473*P*)^2^ + 0.4567*P*] *P* = (*F*_o_^2^ + 2*F*_c_^2^)/3
*S* = 1.06	(Δ/σ)_max_ < 0.001
2551 reflections	Δρ_max_ = 0.30 e Å^−^^3^
151 parameters	Δρ_min_ = −0.51 e Å^−^^3^
Primary atom site location: structure-invariant direct methods	Extinction correction: none

### Special details

**Table:**

Geometry. All e.s.d.'s (except the e.s.d. in the dihedral angle between two l.s. planes) are estimated using the full covariance matrix. The cell e.s.d.'s are taken into account individually in the estimation of e.s.d.'s in distances, angles and torsion angles; correlations between e.s.d.'s in cell parameters are only used when they are defined by crystal symmetry. An approximate (isotropic) treatment of cell e.s.d.'s is used for estimating e.s.d.'s involving l.s. planes.
Refinement. Refinement of *F*^2^ against ALL reflections. The weighted *R*-factor *wR* and goodness of fit *S* are based on *F*^2^, conventional *R*-factors *R* are based on *F*, with *F* set to zero for negative *F*^2^. The threshold expression of *F*^2^ > σ(*F*^2^) is used only for calculating *R*-factors(gt) *etc*. and is not relevant to the choice of reflections for refinement. *R*-factors based on *F*^2^ are statistically about twice as large as those based on *F*, and *R*- factors based on ALL data will be even larger.

### Fractional atomic coordinates and isotropic or equivalent isotropic displacement parameters (Å^2^)

**Table:**

	*x*	*y*	*z*	*U*_iso_*/*U*_eq_	
Cu1	0.5000	0.0000	0.0000	0.03048 (18)	
Cl1	0.18946 (11)	0.17433 (12)	0.43516 (12)	0.0544 (3)	
O1	0.3831 (2)	0.1341 (3)	−0.0575 (3)	0.0410 (6)	
H1W	0.3885	0.2251	0.0267	0.061*	
H2W	0.3032	0.0955	−0.1237	0.061*	
O2	0.6183 (3)	0.2239 (3)	0.1983 (3)	0.0458 (6)	
H3W	0.6168	0.2522	0.2960	0.069*	
H4W	0.6825	0.2950	0.1926	0.069*	
O3	0.3576 (3)	−0.0289 (3)	0.1457 (3)	0.0468 (6)	
H5W	0.3400	0.0621	0.2072	0.070*	
H6W	0.3653	−0.0847	0.1901	0.070*	
O4	0.1965 (3)	0.5031 (3)	0.7782 (3)	0.0481 (6)	
H7W	0.2086	0.4204	0.6934	0.072*	
H8W	0.1057	0.4942	0.7787	0.072*	
O5	0.1485 (3)	0.0517 (4)	0.7004 (4)	0.0741 (9)	
H9W	0.1697	0.0946	0.6432	0.111*	
H10W	0.0599	−0.0029	0.6717	0.111*	
N1	0.3348 (3)	0.7402 (3)	0.2551 (3)	0.0349 (6)	
N2	0.3362 (3)	0.6544 (3)	0.4602 (3)	0.0347 (6)	
N3	0.3419 (3)	0.4512 (3)	0.1727 (3)	0.0339 (6)	
N4	0.1150 (3)	0.5418 (3)	0.2441 (3)	0.0352 (6)	
C1	0.3865 (4)	0.7928 (4)	0.4281 (4)	0.0372 (7)	
H1A	0.3493	0.8884	0.5004	0.045*	
H1B	0.4945	0.8297	0.4544	0.045*	
C2	0.3940 (4)	0.5117 (4)	0.3491 (4)	0.0367 (7)	
H2A	0.3622	0.4193	0.3685	0.044*	
H2B	0.5021	0.5469	0.3747	0.044*	
C3	0.1779 (4)	0.4020 (4)	0.1381 (4)	0.0399 (8)	
H3A	0.1418	0.3631	0.0225	0.048*	
H3B	0.1433	0.3083	0.1550	0.048*	
C4	0.1704 (4)	0.6831 (4)	0.2180 (4)	0.0390 (8)	
H4A	0.1311	0.7774	0.2886	0.047*	
H4B	0.1342	0.6476	0.1034	0.047*	
C5	0.3921 (4)	0.5949 (4)	0.1478 (4)	0.0384 (8)	
H5A	0.5002	0.6301	0.1718	0.046*	
H5B	0.3584	0.5583	0.0324	0.046*	
C6	0.1729 (4)	0.5996 (4)	0.4188 (4)	0.0388 (8)	
H6A	0.1335	0.6931	0.4911	0.047*	
H6B	0.1386	0.5079	0.4386	0.047*	

### Atomic displacement parameters (Å^2^)

**Table:**

	*U*^11^	*U*^22^	*U*^33^	*U*^12^	*U*^13^	*U*^23^
Cu1	0.0393 (3)	0.0273 (3)	0.0255 (3)	0.0104 (2)	0.0087 (2)	0.0133 (2)
Cl1	0.0599 (6)	0.0441 (5)	0.0534 (6)	0.0137 (5)	0.0259 (5)	0.0190 (5)
O1	0.0505 (14)	0.0335 (12)	0.0335 (12)	0.0193 (11)	0.0002 (10)	0.0117 (10)
O2	0.0663 (16)	0.0310 (12)	0.0231 (11)	−0.0071 (11)	0.0028 (11)	0.0093 (10)
O3	0.0699 (17)	0.0442 (14)	0.0551 (15)	0.0328 (13)	0.0383 (13)	0.0372 (13)
O4	0.0416 (14)	0.0439 (14)	0.0435 (14)	0.0008 (11)	0.0087 (11)	0.0151 (12)
O5	0.0623 (18)	0.088 (2)	0.070 (2)	0.0023 (16)	−0.0133 (15)	0.0494 (19)
N1	0.0419 (16)	0.0337 (15)	0.0399 (15)	0.0157 (13)	0.0169 (12)	0.0237 (13)
N2	0.0422 (15)	0.0331 (14)	0.0248 (13)	0.0026 (12)	0.0077 (11)	0.0143 (12)
N3	0.0421 (15)	0.0299 (14)	0.0300 (14)	0.0139 (12)	0.0067 (12)	0.0141 (12)
N4	0.0353 (15)	0.0330 (15)	0.0333 (14)	0.0089 (12)	0.0074 (12)	0.0141 (12)
C1	0.0456 (19)	0.0255 (16)	0.0339 (17)	0.0049 (14)	0.0106 (15)	0.0117 (14)
C2	0.0448 (19)	0.0355 (18)	0.0335 (17)	0.0093 (15)	0.0032 (14)	0.0214 (15)
C3	0.0403 (19)	0.0323 (18)	0.0335 (18)	0.0067 (15)	0.0012 (15)	0.0090 (15)
C4	0.047 (2)	0.0410 (19)	0.0398 (18)	0.0225 (16)	0.0150 (15)	0.0240 (16)
C5	0.047 (2)	0.046 (2)	0.0327 (17)	0.0224 (17)	0.0174 (15)	0.0240 (16)
C6	0.048 (2)	0.0343 (18)	0.0359 (18)	0.0082 (15)	0.0176 (15)	0.0190 (15)

### Geometric parameters (Å, °)

**Table:**

Cu1—O2	2.017 (2)	N2—C2	1.469 (4)
Cu1—O2^i^	2.017 (2)	N2—C1	1.475 (4)
Cu1—O1	2.045 (2)	N3—C3	1.472 (4)
Cu1—O1^i^	2.045 (2)	N3—C2	1.473 (4)
Cu1—O3^i^	2.053 (2)	N3—C5	1.476 (4)
Cu1—O3	2.053 (2)	N4—C3	1.467 (4)
O1—H1W	0.8200	N4—C4	1.472 (4)
O1—H2W	0.8260	N4—C6	1.474 (4)
O2—H3W	0.8254	C1—H1A	0.9700
O2—H4W	0.8330	C1—H1B	0.9700
O3—H5W	0.8200	C2—H2A	0.9700
O3—H6W	0.8246	C2—H2B	0.9700
O4—H7W	0.8304	C3—H3A	0.9700
O4—H8W	0.8351	C3—H3B	0.9700
O5—H9W	0.8312	C4—H4A	0.9700
O5—H10W	0.8289	C4—H4B	0.9700
N1—C1	1.462 (4)	C5—H5A	0.9700
N1—C5	1.473 (4)	C5—H5B	0.9700
N1—C4	1.477 (4)	C6—H6A	0.9700
N2—C6	1.466 (4)	C6—H6B	0.9700
			
O2—Cu1—O2^i^	180	C4—N4—C6	107.7 (2)
O2—Cu1—O1	87.24 (9)	N1—C1—N2	111.9 (2)
O2^i^—Cu1—O1	92.76 (9)	N1—C1—H1A	109.2
O2—Cu1—O1^i^	92.76 (9)	N2—C1—H1A	109.2
O2^i^—Cu1—O1^i^	87.24 (9)	N1—C1—H1B	109.2
O1—Cu1—O1^i^	180	N2—C1—H1B	109.2
O2—Cu1—O3^i^	90.30 (10)	H1A—C1—H1B	107.9
O2^i^—Cu1—O3^i^	89.70 (10)	N2—C2—N3	112.0 (2)
O1—Cu1—O3^i^	86.64 (9)	N2—C2—H2A	109.2
O1^i^—Cu1—O3^i^	93.36 (9)	N3—C2—H2A	109.2
O2—Cu1—O3	89.70 (10)	N2—C2—H2B	109.2
O2^i^—Cu1—O3	90.30 (10)	N3—C2—H2B	109.2
O1—Cu1—O3	93.36 (9)	H2A—C2—H2B	107.9
O1^i^—Cu1—O3	86.64 (9)	N4—C3—N3	112.7 (3)
O3^i^—Cu1—O3	180	N4—C3—H3A	109.1
Cu1—O1—H1W	109.5	N3—C3—H3A	109.1
Cu1—O1—H2W	126.7	N4—C3—H3B	109.1
H1W—O1—H2W	113.2	N3—C3—H3B	109.1
Cu1—O2—H3W	124.5	H3A—C3—H3B	107.8
Cu1—O2—H4W	124.2	N4—C4—N1	112.3 (2)
H3W—O2—H4W	110.9	N4—C4—H4A	109.2
Cu1—O3—H5W	109.5	N1—C4—H4A	109.2
Cu1—O3—H6W	123.5	N4—C4—H4B	109.2
H5W—O3—H6W	113.5	N1—C4—H4B	109.2
H7W—O4—H8W	110.2	H4A—C4—H4B	107.9
H9W—O5—H10W	111.4	N1—C5—N3	112.0 (2)
C1—N1—C5	108.3 (2)	N1—C5—H5A	109.2
C1—N1—C4	108.1 (2)	N3—C5—H5A	109.2
C5—N1—C4	108.3 (3)	N1—C5—H5B	109.2
C6—N2—C2	108.5 (2)	N3—C5—H5B	109.2
C6—N2—C1	108.5 (2)	H5A—C5—H5B	107.9
C2—N2—C1	108.0 (2)	N2—C6—N4	112.1 (2)
C3—N3—C2	108.0 (3)	N2—C6—H6A	109.2
C3—N3—C5	108.1 (2)	N4—C6—H6A	109.2
C2—N3—C5	107.7 (2)	N2—C6—H6B	109.2
C3—N4—C4	108.1 (3)	N4—C6—H6B	109.2
C3—N4—C6	107.9 (2)	H6A—C6—H6B	107.9
			
C5—N1—C1—N2	−58.7 (3)	C3—N4—C4—N1	−57.9 (3)
C4—N1—C1—N2	58.3 (3)	C6—N4—C4—N1	58.4 (3)
C6—N2—C1—N1	−58.5 (3)	C1—N1—C4—N4	−58.8 (3)
C2—N2—C1—N1	58.9 (3)	C5—N1—C4—N4	58.3 (3)
C6—N2—C2—N3	58.4 (3)	C1—N1—C5—N3	58.7 (3)
C1—N2—C2—N3	−59.0 (3)	C4—N1—C5—N3	−58.2 (3)
C3—N3—C2—N2	−57.7 (3)	C3—N3—C5—N1	58.0 (3)
C5—N3—C2—N2	58.8 (3)	C2—N3—C5—N1	−58.5 (3)
C4—N4—C3—N3	58.1 (3)	C2—N2—C6—N4	−58.6 (3)
C6—N4—C3—N3	−58.2 (3)	C1—N2—C6—N4	58.5 (3)
C2—N3—C3—N4	58.1 (3)	C3—N4—C6—N2	58.2 (3)
C5—N3—C3—N4	−58.2 (3)	C4—N4—C6—N2	−58.3 (3)

Symmetry codes: (i) −*x*+1, −*y*, −*z*.

### Hydrogen-bond geometry (Å, °)

**Table:**

*D*—H···*A*	*D*—H	H···*A*	*D*···*A*	*D*—H···*A*
O1—H1W···N3	0.82	2.04	2.814 (3)	158
O1—H2W···O5^ii^	0.83	1.94	2.734 (3)	162
O2—H3W···N2^iii^	0.83	1.99	2.800 (3)	167
O2—H4W···O4^iii^	0.83	1.89	2.700 (3)	165
O3—H5W···Cl1	0.82	2.54	3.190 (2)	137
O3—H6W···N1^iv^	0.82	2.00	2.805 (3)	165
O4—H7W···Cl1	0.83	2.35	3.170 (3)	168
O4—H8W···N4^v^	0.84	2.00	2.829 (4)	174
O5—H9W···Cl1	0.83	2.43	3.245 (3)	169
O5—H10W···Cl1^vi^	0.83	2.37	3.200 (3)	175

Symmetry codes: (ii) *x*, *y*, *z*−1; (iii) −*x*+1, −*y*+1, −*z*+1; (iv) *x*, *y*−1, *z*; (v) −*x*, −*y*+1, −*z*+1; (vi) −*x*, −*y*, −*z*+1.
